# A non-linear data mining parameter selection algorithm for continuous variables

**DOI:** 10.1371/journal.pone.0187676

**Published:** 2017-11-13

**Authors:** Peyman Tavallali, Marianne Razavi, Sean Brady

**Affiliations:** 1 Division of Engineering and Applied Sciences, California Institute of Technology, Pasadena, California, United States of America; 2 Principium Consulting, LLC, Pasadena, California, United States of America; Jaypee University of Information Technology, INDIA

## Abstract

In this article, we propose a new data mining algorithm, by which one can both capture the non-linearity in data and also find the best subset model. To produce an enhanced subset of the original variables, a preferred selection method should have the potential of adding a supplementary level of regression analysis that would capture complex relationships in the data via mathematical transformation of the predictors and exploration of synergistic effects of combined variables. The method that we present here has the potential to produce an optimal subset of variables, rendering the overall process of model selection more efficient. This algorithm introduces interpretable parameters by transforming the original inputs and also a faithful fit to the data. The core objective of this paper is to introduce a new estimation technique for the classical least square regression framework. This new automatic variable transformation and model selection method could offer an optimal and stable model that minimizes the mean square error and variability, while combining all possible subset selection methodology with the inclusion variable transformations and interactions. Moreover, this method controls multicollinearity, leading to an optimal set of explanatory variables.

## Introduction

It happens often that the physical or mathematical model behind an experiment or dataset is not known. Hence, model selection (sometimes called subset selection) becomes an important feature during the data analysis endeavor. The methodology of selecting the best model from a set of inputs has constantly been examined by many authors [1]. Identifying the best subset among many variables is the most difficult part of this effort. The latter is exacerbated as the number of possible subsets grows exponentially, if the number of variables (parameters) grows linearly. Furthermore, there is also a chance that the original input parameters to a model do not convey enough information. Some transformations of the original parameters, and specifically interactions between them, are needed to make the data more available for information extraction.

In other words, in a supervised learning terminology, there is a long and unpaved journey between the *inputs* (also called *predictors, features* or *independent variables*) and the *outputs* (also called *responses* or *dependent variables*). Thus, the difficulty is not only embedded in picking the right machine learning algorithm for the problem at hand, but also in picking proper transformations and interactions of the inputs or their subsets. There are different methods capable of addressing transformations and subset selection. However, to the best of our knowledge, none of these methods solves both issues simultaneously.

In our discussions in this paper, we denote the vectorial form of an input variable *x* by an *N* × 1 vector **x** as a collection of *N* observations. The assembly of *p* such inputs and an intercept is denoted by an *N* × (*p* + 1) matrix **X** = (**1**, **x**_1_, **x**_2_, …, **x**_*p*_). The vectorial form of the output *y* is denoted by an *N* × 1 vector **Y**. For example, based on this description, a linear model is defined as

$$\begin{array}{c} Y=X\beta +\varepsilon , \end{array}$$ (1)

where *ε* is the *N* × 1 noise vector, and *β* = (*β*_0_, *β*_1_, …, *β*_*p*_)^*T*^ is a (*p* + 1) × 1 vector of coefficients with the first element *β*_0_ as the *intercept* (or *bias*) of the model. In what follows next, we review a series of methods and algorithms that are used to find some subset(s) of the inputs that could possibly relate the inputs to outputs in an efficient way.

### Subset selection

There are currently various methods for selecting predictors, such as the traditional best subset selection, forward selection, backward selection and stepwise selection methods [1, 2]. In general, the best subset procedure finds for each *k* ∈ {1, 2, …, *p*}, the subset of inputs of size *k* that minimizes the Residual Sum of Squares (RSS) [3–6]. There are fast algorithms optimizing the search [7]. However, searching through all possible subsets could become laborious as *p* increases.

A number of automatic subset selection methods seek a subset of all inputs, that is as close as possible to the best subset method [1]. These methods select a subset of predictors by an automated algorithm that meets a predefined criterion, such as the level of significance (set by the analyst). For example, the forward selection method [1] starts with no predictors in the model. It then adds predictors one at a time until no available predictors can contribute significantly to the response variable. Once a predictor is included in the model, it remains there. On the other hand, the backward elimination technique [1] works in the opposite direction and begins with all the existing predictors in the model, then discards them one after another until all remaining predictors contribute significantly to the response variable. Stepwise subset selection [8] is a mixture of the forward and backward selection methods. It modifies the forward selection approach in that variables already in the model do not always remain in the model. Indeed, after each step in which a variable is added, all variables in the model are reevaluated via their partial *F* or *t* statistics and any non-significant variable is removed from the model. The stepwise regression requires two cutoff values for significance: one for adding variables and one for discarding variables. In general, the probability threshold for adding variables should be smaller than the probability threshold for eliminating variables [1].

Subset selection methods are usually based on targeting models with the largest $R_{adj}^{2}$, or in other words smallest Root Mean Square Error (RMSE). However, there are other methods in which the selection model is based on Mallow’s *C*_*p*_ [9–12]. These criteria highlight different aspects of the regression model. As a results, they can lead to models that are completely different from each other and yet not optimal.

Unfortunately, none of these subset selection methods address the issue of multicollinearity.

### Ridge regression

There are also other issues regarding the traditional subset selection regression methods. They could lead to models that are unreliable for prediction because of over-fitting issues. More specifically, they could generate models that have variables displaying a high degree of multicollinearity. Such methods can lead to *R*^2^ values that are biased and yield to confidence limits that are far too narrow or wide. Moreover, the selection criterion primarily relies on the correlation between the predictor(s) and the dependent variable. Thus, these methods (e.g. Stepwise method [13]) do not take into consideration the correlation within the predictors themselves. The latter is a source of multicollinearity that is not addressed automatically by these mentioned methods [13].

Indeed, when collinearity among the predictors exists, the variance of the coefficients is inflated, rendering the overall regression equation unstable. To address this issue, a number of *penalized regression* or *shrinkage* approaches are available. For example, the Ridge method tries to eliminate the multicollinearity by imposing a penalty on the size of the regression coefficients [2]. Indeed, a model is fitted with all the predictors, however, the estimated coefficients are shrunken towards zero relative to the least squared estimates. Therefore, biased estimators of regression coefficients are obtained, reducing the variance and thus leading to a more stable equation.

Solving for *β* in Eq (1) using the Least Squares (LS) method would be equivalent to solving

$$\begin{array}{c} {\hat{\beta }}^{LS}=\underset{\beta }{\arg\min}{∥Y-X\beta ∥}_{2}^{2}. \end{array}$$ (2)

Here, ${∥x∥}_{2}={\left(\sum {|x_{j}|}^{2}\right)}^{\frac{1}{2}}$ is the *L*_2_ norm of **x**. Ridge regression, on the other hand, places a constraint on the estimator *β* in order to minimize a penalized sum of squares [14, 15]

$$\begin{array}{c} {\hat{\beta }}^{ridge}=\underset{\beta }{\arg\min}{∥Y-X\beta ∥}_{2}^{2}+\lambda {∥\beta ∥}_{2}^{2}. \end{array}$$ (3)

The complexity parameter λ ⩾ 0 controls the amount of shrinkage. Large values of this parameter would result in a large shrinkage. The value of the constant λ is predefined by the analyst and is usually selected in order to stabilize the ridge estimators, producing an improved equation with a smaller RMSE compared to the least-squares estimates. One weakness of the Ridge method is that it does not select variables. Indeed, unlike the subset selection method, it includes all of the predictors in the final model with shrunken coefficients. The other weakness is that multicollinearity is not fully addressed. In fact, the Ridge estimate of variables in (3) only shrinks the coefficients even for the inputs with multicollinearity. However, the Ridge Method does not fix multicollinearity, it only alleviates it. This issue has been shown and addressed in [16].

### Lasso

To obtain variable selection procedures, there are shrinkage methods available such as Least Absolute Shrinkage and Selection Operator (Lasso), where the penalty involves the sum of the absolute values of the coefficients *β* excluding the intercept [17]. Lasso is closely related to sparse optimization found in works by Candes and Tao [18]. Taking *β*^−^ = (*β*_1_, …, *β*_*p*_)^*T*^, the Lasso method can be presented as the following optimization problem

$$\begin{array}{c} {\hat{\beta }}^{Lasso}=\underset{\beta }{\arg\min}{∥Y-X\beta ∥}_{2}^{2}+\lambda {∥\beta^{-}∥}_{1}, \end{array}$$ (4)

where ${∥\beta^{-}∥}_{1}=\sum_{1}^{p}\left|\beta_{j}\right|$ is the *L*_1_ norm of *β*^−^ and λ > 0. The advantage of Lasso is that much like the best subset selection method, it performs variable selection.

The parameter λ is usually selected by cross validation. For a small λ, the result is equal to the least squares estimates. As the value of λ augments, shrinkage happens in such a way that only a sparse number of variables having an active role in the final model would show up. Thus, Lasso is a combination of both shrinkage and variables selection.

### LAR

Least Angle Regression (LAR) is a new model of automatic subset selection based on a modified version of forward procedure [19]. The LAR method follows an algorithmic procedure: First, the independent variables are standardized in order to obtain a mean zero. At this stage, the *β* coefficients are all equal to zero. Then the predictor that most correlates to the response variable is selected; its coefficient is then shifted from zero towards its least squares value. Now, once a second predictor becomes as correlated with the existing residual as the first predictor, the procedure is paused. The second predictor is then added to the model. This procedure then continues until all desired predictors are included in the model, leading to a full least-squares fit.

The method of Least Angle Regression with Lasso modification is very similar to the above procedure, however it includes an extra step: if a coefficient approaches zero, LAR excludes its predictor from the model and recalculates the joint least squares path [2]. LAR methods and its variations are better subset selector algorithms compared to most of the subset selection methods.

### Dantzig

Another selection approach is the Dantzig selector [20], which can be formulated as

$$\begin{array}{c} \min_{\beta }{∥X^{T}\left(Y-X\beta \right)∥}_{\infty } \end{array}$$ (5)

subject to ‖*β*‖_1_ ≤ *t*. Here, ‖.‖_∞_ is the *L*_∞_ norm, that is the maximum of its argument. The objective of this method is to minimize the maximum inner product of the existing residual with all the independent variables. This approach has the capacity of recovering an underlying sparse coefficient vector.

### Knockoff filter

This method is recently introduced as a new variable selection method to control the false discovery rate (FDR) [21], for linear models. For a selected subset of variable indices $\hat{S}$, the FDR is formally defined as

$$\begin{array}{c} FDR=E(\frac{\#\{j|\beta_{j}=0,\;j\in \hat{S}\}}{\max(\#\left\{j|j\in \hat{S}\right\},1)}). \end{array}$$ (6)

It is also well-suited for high-dimensional linear models in which the number of features are more than the number of data points. This method is capable of being combined with different methods, such as Lasso explained above, to perform a more reliable variable selection in the context of controlling the FDR.

### PCR

Lastly, Principal Component Regression (PCR) is a method that involves an orthogonal transformation to address multicollinearity [2, 22, 23]. This approach is closely related to the Singular Value Decomposition (SVD) method [24]. PCR applies dimensionality reduction and decreases multicollinearity by using a subset of the principal components in the model [2]. PCR is one of very few methods that tries to eliminate multicollinearity with linear transformations and, at the same time, perform a regression.

The various approaches described so far aim to select the best set of relevant variables from an original set. With the exception of the PCR method, in which there are linear transformations, variables transformations are not incorporated among predictors in any of the methods mentioned above. These traditional methods do not offer the option of automatic variable transformation to address polynomial curvilinear relationships. No non-linear interpretable interaction of the predictors is available in them. An analyst usually needs to manually apply polynomial, logarithmic, square-root and interaction-between-variables transformations in order to address non-linearity of the data.

#### Non-Linear transformation

There are a number of non-linear transformation procedures currently available such as Box-Cox or Box-Tidwell [25, 26]. These methods are relatively efficient in finding the dependent and independent variables transformations. In Box-Tidwell method [26], independent variables are transformed using a recursive Newton algorithm. As a result, it becomes susceptible to round-off errors which would in turn result in unstable and improper transformations [1]. Despite the relative success of these methods, there is no automatic variable selection embodiment with them.

Artificial Neural Networks (ANN) are the current state of the art method in transformations and capturing non-linearity [2, 27]. ANN is a machine learning method that finds some non- linear transformations of the inputs using layers of nodes. One recent exemplary example is the Deep Neural Networks (DNN) used in speech recognition [28]. Despite the efficient performance in capturing the non-linearity of the data, the model itself is not comprehensible particularly if there is a physical component to the data that one needs to interpret or understand. In other words, ANN is a perfect black box model, but not a good interpretable medium for understanding physical and mathematical mechanism(s) behind the observed data.

#### Subset selection and transformation

As mentioned earlier, only the PCR method performs linear transformations automatically, and also picks variables. However, PCR is not enough when non-linearity is present. On the other hand, ANN has the best capability in capturing non-linearities, but acts like a black box and does not lend insight into the physical and mathematical mechanism(s) behind the observed data.

To produce an enhanced subset of the original variables, an effective selection method should have the potential of adding a supplementary level of regression analysis that would capture complex relationships in the data via mathematical transformation of the predictors and exploration of synergistic effects of combined variables in an interpretable fashion. The method that we present here has the potential to produce an optimal subset of variables, which is even interpretable in the presence of non-linear interaction between the inputs, resulting in a more efficient overall process of model selection.

The core objective of this paper is to introduce a new estimation technique for the classical least square regression framework. This new automatic variable transformation and model selection method could offer an efficient and stable model that minimizes the mean square error and variability, while combining all possible subset selection methodologies and including variable transformations and interaction. Moreover, this novel method controls multicollinearity, leading to an optimal set of explanatory variables. The final model is also easy to interpret. In other words, we will depict a method that tries to address variable selection, interpretation, non-linear interaction and transformation at the same time.

## Materials and methods

### Problem definition

We assume T to be the set of all transformations on a given set of inputs {*x*_*i*_}, for *i* ∈ {1, …, *p*} and $x_{i}\in R$. One possible formulation, to find the best subset and transformation estimating a dependent variable y∈R, can be expressed as

$$\begin{array}{c} \begin{array}{cc} minimize & ∥y-f({\left\{x_{j}\right\}}_{j\in \Omega })∥\\ f\in T & \\ \Omega \subset P(\{1,\ldots ,p\}) \end{array} \end{array}$$ (7)

Here, one desirable candidate for the norm ‖.‖ could be the *L*_2_ norm, since the purpose is regression. Also, P(.) is the power set. This is an NP hard problem. As a result, we need to find approximations of this problem to make it traceable.

In the first step, we confine ourselves to a set of certain functions in T that are easy to interpret from a casual physical perspective. We call this set F. For example we could pick only the polynomial transformations. Consequently, the set of all transformed variables would be

$$\begin{array}{c} Z=\{F\left({\left\{x_{i}\right\}}_{i\in \{1,\ldots ,p\}}\right)\}. \end{array}$$ (8)

This step would reduce the search space for (7). However, there are sources of redundancy which we could minimize or eliminate. Knowing this, the next step could be to pick transformed variables that have a significant absolute value correlation *ρ*_*zy*_ with the output *y*. This set can be expressed as

$$\begin{array}{c} Z^{\delta }=\left\{z\in Z|\rho_{zy}\geq \delta ,\;\delta >0\right\}. \end{array}$$ (9)

Also, there is a chance that many of the elements in *Z*^*δ*^ are strongly correlated with each other. Later, this could be a serious source of multi-collinearity. So, we could further trim *Z*^*δ*^ by only picking the most correlated variables to the output among two correlated variables. This would reduce the set *Z*^*δ*^ to

$$\begin{array}{ccc} Z^{r} & = & \{z\in Z^{\delta }|z=\underset{\alpha ,\beta \in Z^{\delta }}{\arg\max}\left(\rho_{\alpha y},\rho_{\beta y}\right)\;s.t.\;\rho_{\alpha \beta }>\varsigma >0\}\cup \\ & & \{z\in Z^{\delta }|\forall \alpha \in Z^{\delta },\;\alpha \neq z,\;\varsigma >0\Rightarrow \rho_{\alpha z}<\varsigma \}. \end{array}$$ (10)

Here, *ρ*_*αβ*_ is the absolute value correlation between *α* and *β*.

At this stage, using (8)-(10), and considering that we are looking for a linear estimator among these reduced transformations, the optimization problem (7) would become

$$\begin{array}{c} \underset{{\left\{\beta_{i}\right\}}_{i\in ℸ},ℸ\subset \left\{1,\ldots ,\right|Z^{r}\left|\right\}}{minimize}\;{∥y-\sum_{i\in ℸ}\beta_{i}z_{i}^{r}∥}_{2}. \end{array}$$ (11)

Here, $Z^{r}={\left\{z_{i}^{r}\right\}}_{i\in \{1,\ldots ,|Z^{r}|\}}$ and |*Z*^*r*^| is the cardinality of *Z*^*r*^. The optimization problem (11) is nothing but a subset selection model and could be approximated by any methods of subset selection [1, 2]. Hence, we now have a model (11) that not only takes care of some desirable interpretable transformations, but also extracts the most meaningful set of parameters.

#### Note

As we intend to provide a data mining method rather than a pure statistical one, the easy interpretation would act as a constraint on the types of transformations in (7). For example, in a medical investigation, the investigator is mainly looking for basic algebraic interactions between the inputs which can provide physiological view of the system under scrutiny. Hence, the non-linear transformations and interaction between terms must be as basic as possible, such as exponents, logarithms, multiplications and etc. On the other hand, a linear model, like (11), should be used to keep the interpretability of the model intact providing a robust and accurate model. By this formulation, we are trying to deploy an interpretable and accurate data mining model, instead of a black-box pure statistical learning method. Our effort is not to compete with statistical learning methods, but to provide an easy and a faithful-fit data mining method. In the next section, we are going to discuss our methodology in more practical detail.

### Methodology

As mentioned before, we are looking for transformations that are easy to interpret. There are four main transformation categories of this type capturing the non-linearity in a data set [2]. These transformations are as follow but not limited to

1. Logarithmic transformation of a positive variable; i.e. log *x*_*j*_,
2. Square-root transformation of a positive variable; i.e. $\sqrt{x_{j}}$,
3. Integer powers up to a certain amount α∈N; i.e. $\left\{\frac{1}{x_{j}^{\alpha }},\frac{1}{x_{j}^{\alpha -1}},\ldots ,x_{j}^{\alpha -1},x_{j}^{\alpha }\right\}$,
4. Interactions between terms created in 1-3 up to a certain amount *M*; e.g., for *M* = 2, possible candidates would be $\frac{1}{x_{i}}$, $\frac{x_{i}^{2}}{x_{j}}$, *x*_*i*_, $x_{i}^{2}x_{j}^{2}$, $x_{i}^{2}{\left(\log x_{j}\right)}^{2}$ and $\frac{\sqrt{x_{i}}}{x_{j}}$.

We are going to use this set of transformations, namely $F({\left\{x_{j}\right\}}_{j\in \Omega };\alpha ,M)$, for the rest of this paper. After the construction of these interpretable interactions transformations, one can start to look for the best model, for **Y**, among the set of all transformations 1-4. Here, $Y\in R^{N}$ is the vector form of the output *y*.

Denoting the set of variables created by transformations 1-4 as **Z**, which is the matrix form of *Z* in (8), we are looking for the best model

$$\begin{array}{c} Y=Z\beta_{z}+\varepsilon , \end{array}$$ (12)

where some elements of *β*_*z*_ are zero. We note that we could further equip our algorithm with Standardized Regression (similar to the first step of the LAR method) to diminish the possibility of a numerically ill-conditioned variable matrix *Z*. In fact, some elements of *β*_*z*_ are zero since there is a chance that some columns of **Z** are linearly dependent or that they do not contribute to any correlation with **Y**. We can address these two issues, by a modified dictionary search [17] algorithm as follows. This part stands out for (9) and (10).

- Any column of **Z** that has a non-significant correlation (less than *δ*) with **Y** can be discarded; see (9).
- Any two columns of **Z** that have a high correlation to each other (greater than *ς*) are redundant columns. Between these two columns, the one that has a higher correlation with **Y** is picked and the other is discarded; see (10).

As a result of this methodology, we can now solve model (12) for only a reduced matrix. We denote this reduced matrix as **Z**^*r*^ and its corresponding vector of coefficients as $\beta_{z}^{r}$.

The final task is to find the best subset of the columns in **Z**^*r*^ to model the data in **Y**. The latter can be done by any method of subset selection including the best subset selection method [1]. The subset selection method that we have used in our implementation is based on targeting models with the largest $R_{adj}^{2}$, or in other words smallest RMSE. As a reference point, we call our methodology the Parameter Selection Algorithm.

### Parameter selection algorithm

The goal of the Parameter Selection Algorithm is to find the best interpretable model on the original observed variables **X**, from a set of basic transformations, estimating **Y**. Our method is summarized in Algorithm 1. Step 1 of this algorithm is input specification. Step 2 is where the dictionary of transformations and interactions is made. Steps 3 and 4 correspond to the elimination of columns of the dictionary which involve either a non-significant correlation to the output or multicollinearity between its elements. Step 5 is where the best model is finally found, subject to the constraint that the final set of variables has a Variation Inflation Factor (*VIF*) less than 10. *VIF* elements are the main diagonal values of the inverse of the multiplication of the input matrix transposed with the input matrix. For example if **X** is the input, then **C** = (**X**^*T*^ **X**)^−1^ and *VIF*_*j*_ = *C*_*jj*_ [1]. Although we eliminate similar-looking variables in step 4, checking for the *VIF* [29] is a necessary condition to make sure that no multicollinearity is introduced into the final model. In practice, step 5 can be solved by maximizing the $R_{adj}^{2}$ among all possible subsets of the variables in **Z**^*r*^ [1].

Steps 2, 3 and 5 in this algorithm can be made parallel to decrease the computational time of the method. To our best knowledge, Algorithm 1 is the first linear data mining method that performs both variable transformation and model selection while adding interaction terms and also preventing multi-collinearity, in one package.

The hyper-parameters *δ* and *ς* are important factors in controlling the speed of convergence of the Parameter Selection Algorithm. In Algorithm 1, the smaller the value of *δ* (similarly, the larger the value of *ς*), the bigger the space of search in step 5. As a result, the speed of convergence would depend greatly on these two parameters.

**Algorithm 1** Parameter Selection Algorithm

 1. Inputs to the algorithm: **X**, **Y**, *α*, *M*, *δ*, *ς*.

 2. Construct the matrix of transformations Z=F(X;α,M).

 3. Construct the matrix **Z**^*δ*^ from **Z**.

 4. Construct the matrix **Z**^*r*^ from **Z**^*δ*^.

 5. Solve $\underset{{\left\{\beta_{i}\right\}}_{i\in ℸ}}{minimize}{∥Y-\sum_{i\in ℸ}\beta_{i}z_{i}^{r}∥}_{2}$ subject to *VIF* ⩽ 10.

#### Candidates for *ς* and *δ*

The hyper-parameter *δ* is straightforward to settle. Most of the contribution of a model comes from variables having a high univariate correlation coefficient with the output. As a result, we could discard variables with smaller contributions. Here, small is measured with respect to the highest absolute value univariate correlation coefficient with the output. Usually, an absolute value univariate correlation coefficient of 0.5, and above, is considered to be high [30]. This is 50% of the maximum allowed absolute correlation of 1. Hence, from a conservative perspective, we could set *δ* to be half of the maximum highest absolute value univariate correlation coefficient among all variables. We call this the default value of *δ*.

On the other hand, the hyper-parameter *ς* can be characterized with the *VIF* concept. Each element of the *VIF* vector can be expressed as

$$\begin{array}{c} VIF_{j}=\frac{1}{1-R_{j}^{2}}. \end{array}$$ (13)

Here, $R_{j}^{2}$ is the multiple *R*^2^ for the regression of **x**_*j*_ against other inputs. Hence, if we want two inputs to have a small correlation with each other, we must have a possible *VIF* between them to be less than 10. This would impose an *R*^2^ = 0.9 between those variables. Hence, a correlation of ∼0.95 would say if two inputs are highly correlated or not. On the other hand, we know that if we set the independence limit ς=0.95, we would construct a huge dictionary of inputs when transformations are available. To have a balance between the two, our recommendation is ς=0.80.

### Synthetic examples

In this section, we provide a few synthetic examples using Parameter Selection Algorithm. In the following examples, we try to show that the algorithm that we have proposed is capable of finding the non-linear transformations in a model.

**Fig 1:**
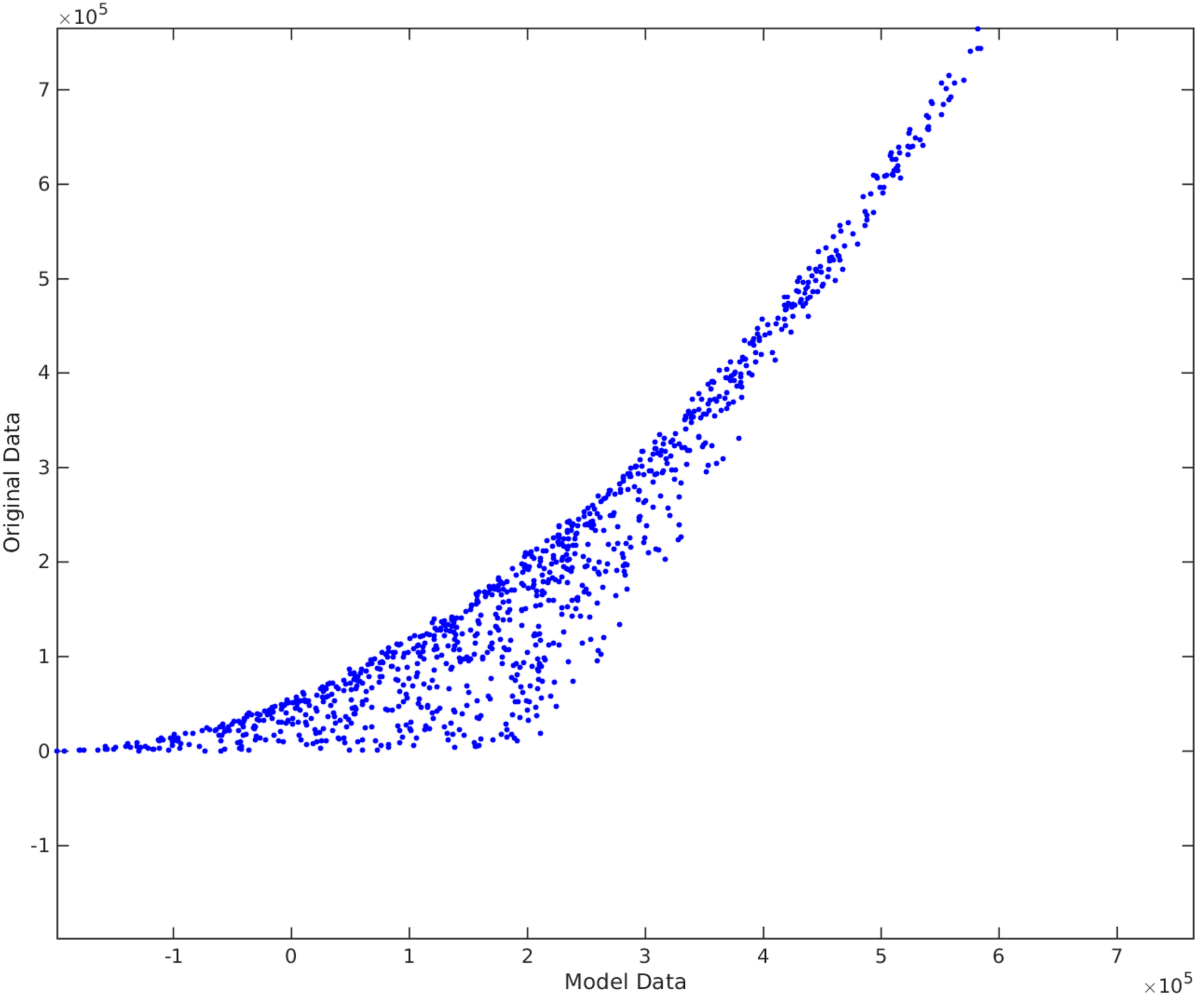
Traditional best subset selection method applied on *y* = 120 + 80*x*_1_*x*_3_. The horizontal axis shows the model found by the best subset selection method. The vertical axis shows the output *y*.

**Fig 2:**
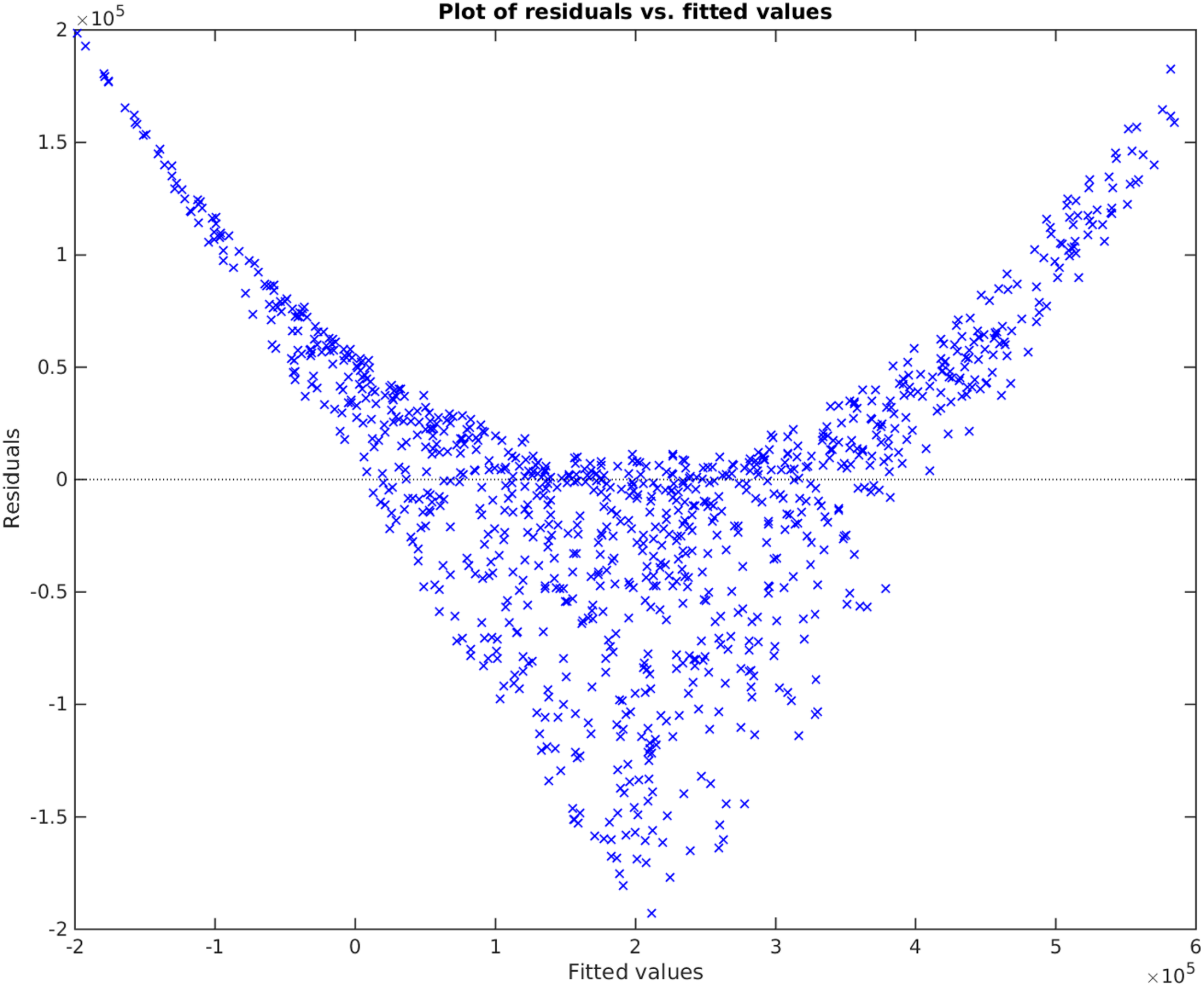
Residual plot of the best subset selection method applied on *y* = 120 + 80*x*_1_*x*_3_.

**Fig 3:**
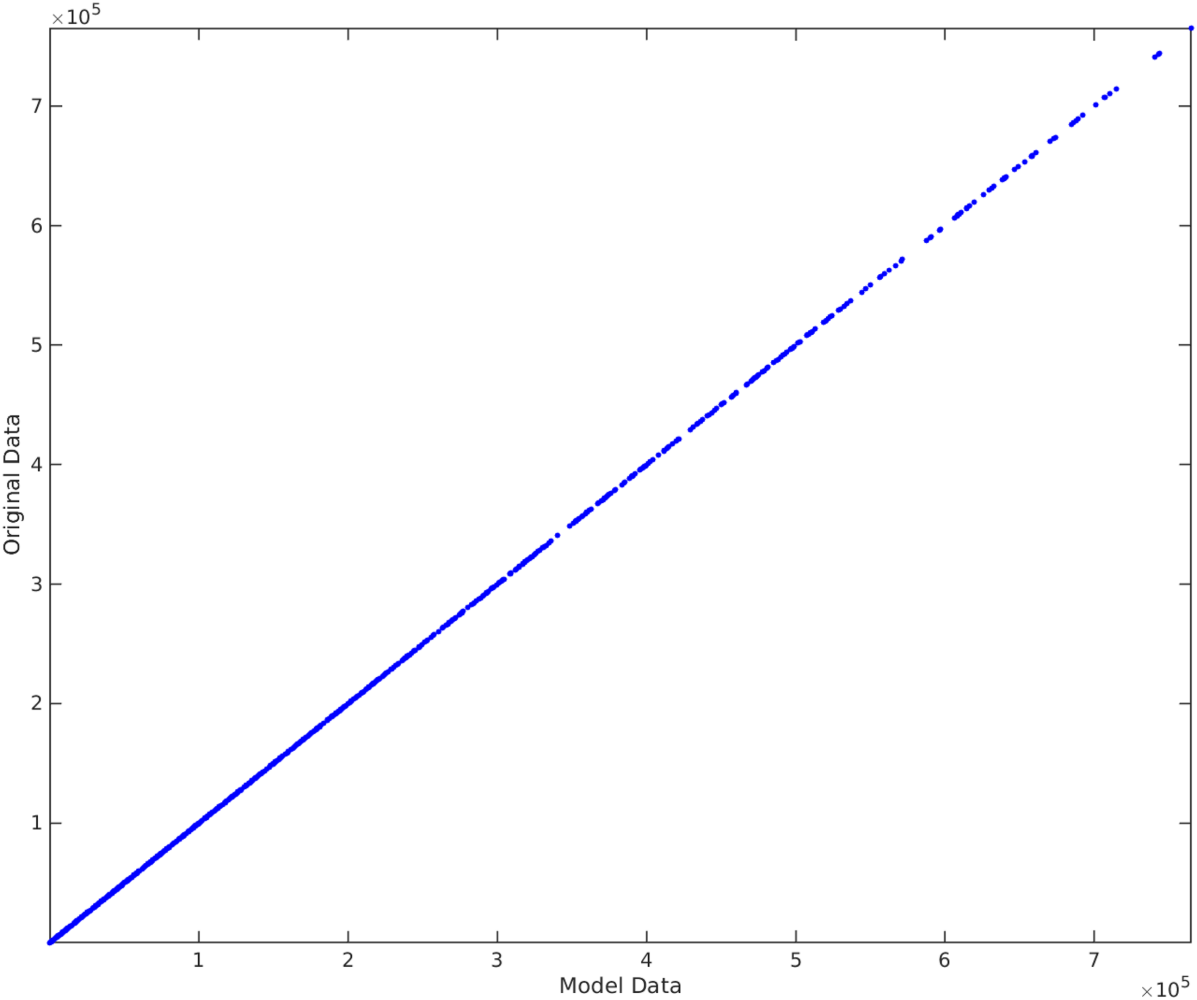
Algorithm 1 applied on *y* = 120 + 80*x*_1_*x*_3_. The horizontal axis shows the model found by our proposed method. The vertical axis shows the output *y*.

**Fig 4:**
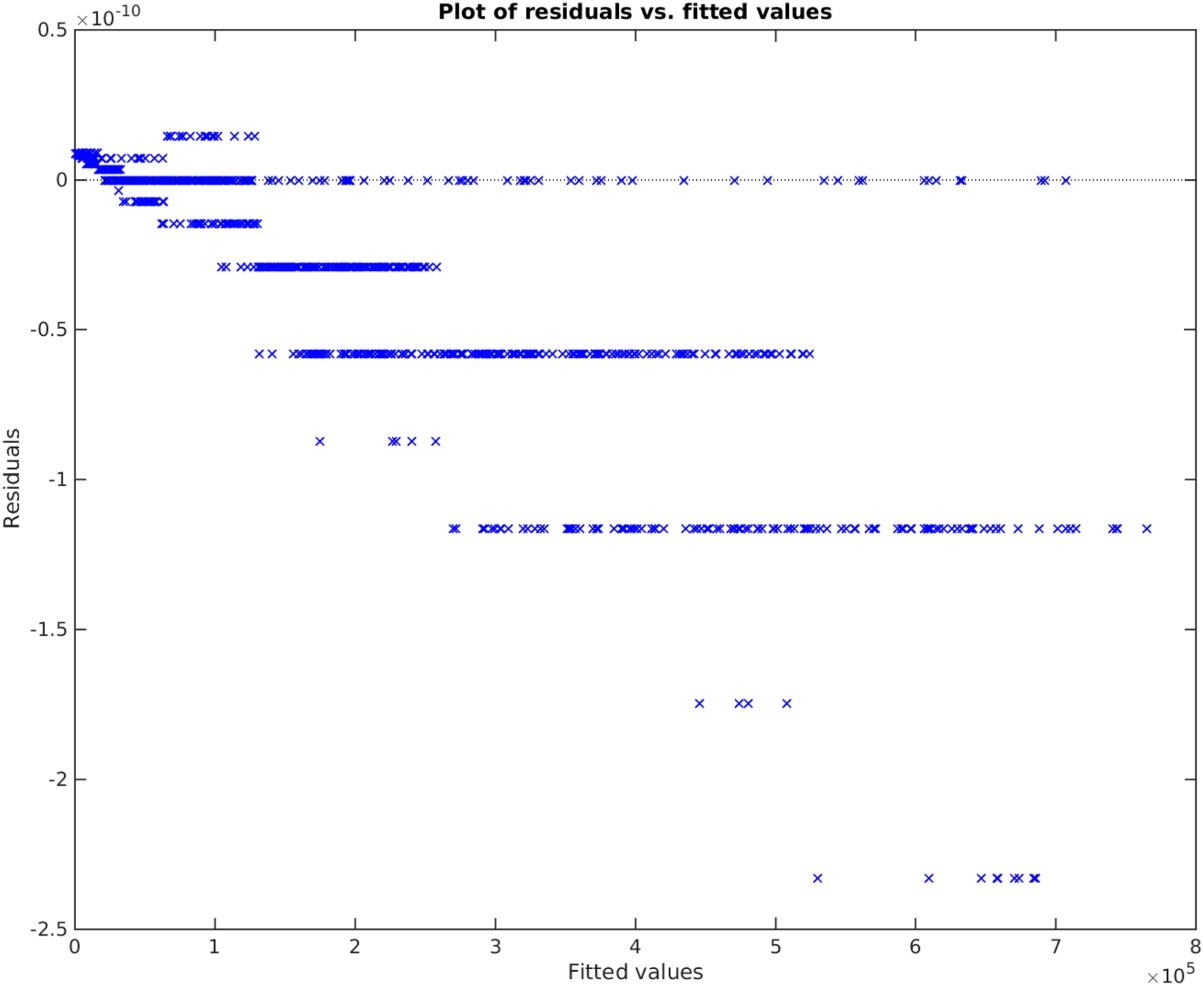
Residual plot of our proposed method applied on *y* = 120 + 80*x*_1_*x*_3_. Note that the vertical axis is of the order 10^−10^. The error perceived here is due to floating point and rounding error.

**Example**. Taking *x*_1_, *x*_2_, *x*_3_ to be independent uniformly distributed random variables between 0 and 100, we sampled 1000 data points and then created the non-linear functional *y* = 120 + 80*x*_1_*x*_3_. We take the original input matrix **X** to be composed of all *x*_1_, *x*_2_, and *x*_3_. Using the traditional best subset selection [7], accompanied with a control over *VIF* not to get above 10, we get the results shown in Fig 1. From this figure, it is clear that the best subset selection model is not capable of capturing the correct non-linearity in the model. The heteroscedasticity of the residual plot can be seen in Fig 2. The found best subset of parameters is {*x*_1_, *x*_2_, *x*_3_}. On the other hand, if Algorithm 1 is used, with a strict choice of ς=0.5 and the default value of *δ*, the non-linearity is captured completely by our method (See Figs 3 and 4). The subset of parameters found by our method is the model non-linear parameter {*x*_1_*x*_3_}.

**Example**. If *χ* is a uniform random random variable between 0 and 1, we set

$$\begin{array}{ccc} x_{1} & = & 100\chi ,\\ x_{2} & = & \chi +0.1,\\ x_{3} & = & 100\chi . \end{array}$$ (14)

**Fig 5:**
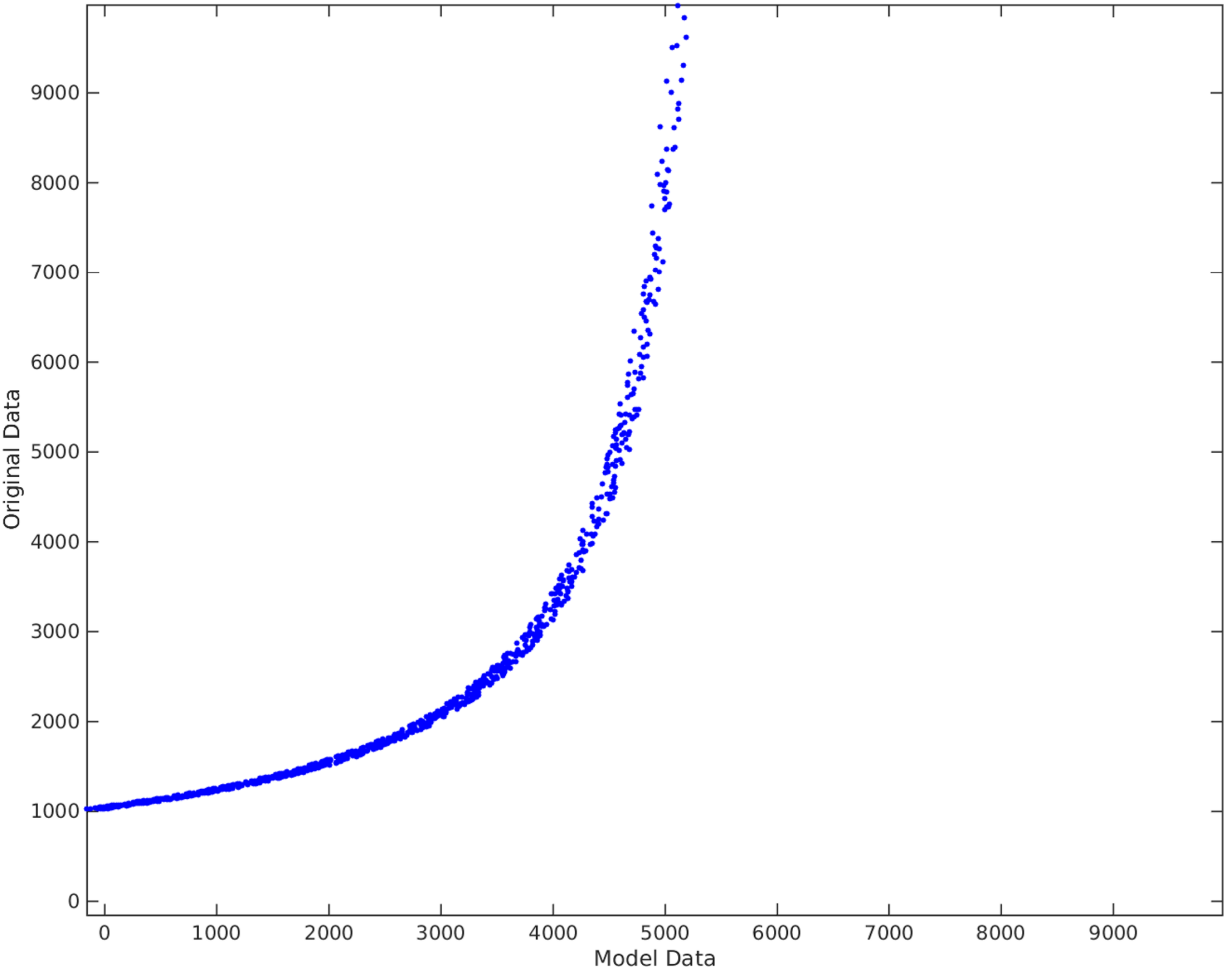
Traditional best subset selection method applied on $y=120+\frac{1000}{x_{2}}$. The horizontal axis shows the model found by the best subset selection method. The vertical axis shows the output *y*.

**Fig 6:**
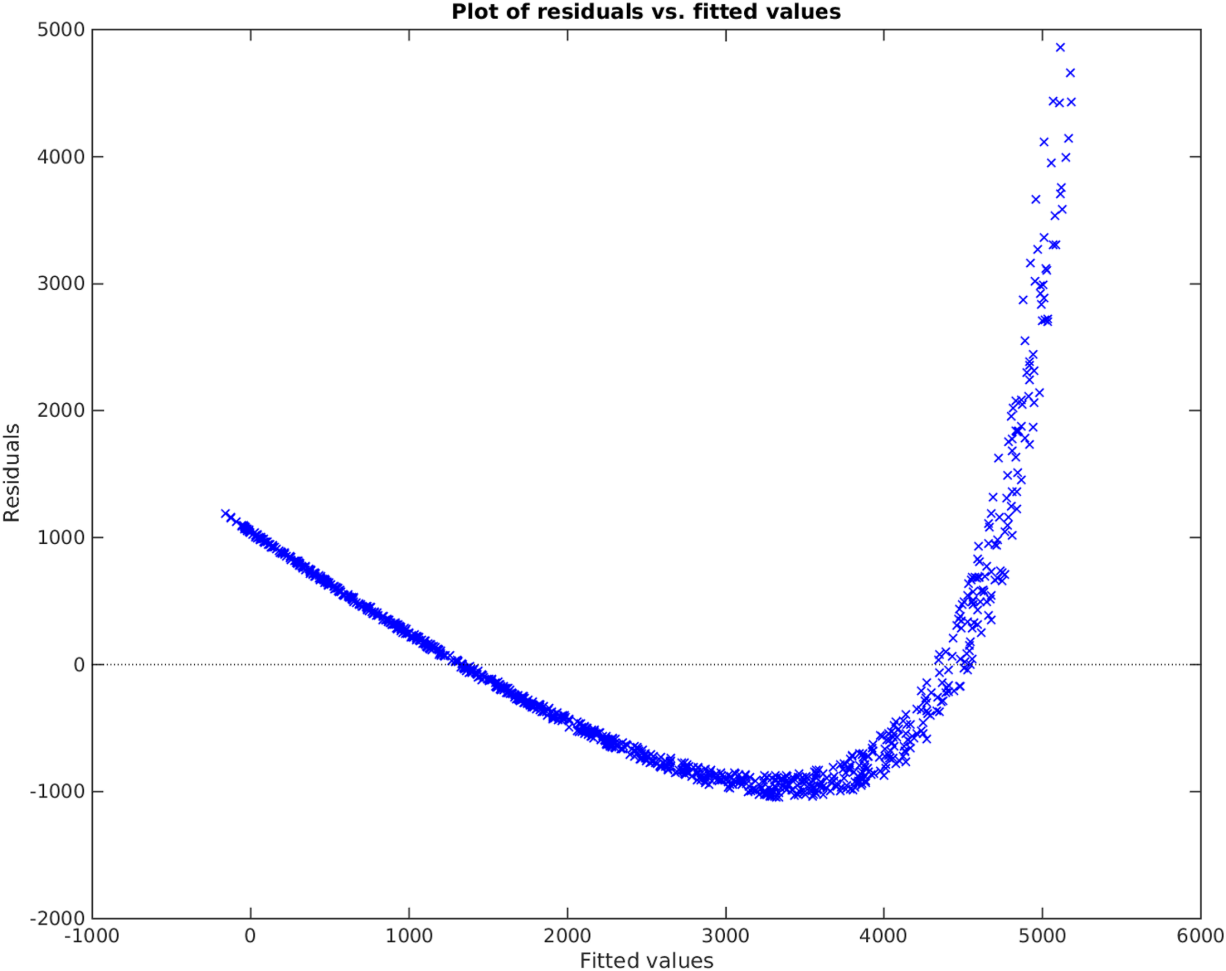
Residual plot of the best subset selection method applied on $y=120+\frac{1000}{x_{2}}$.

**Fig 7:**
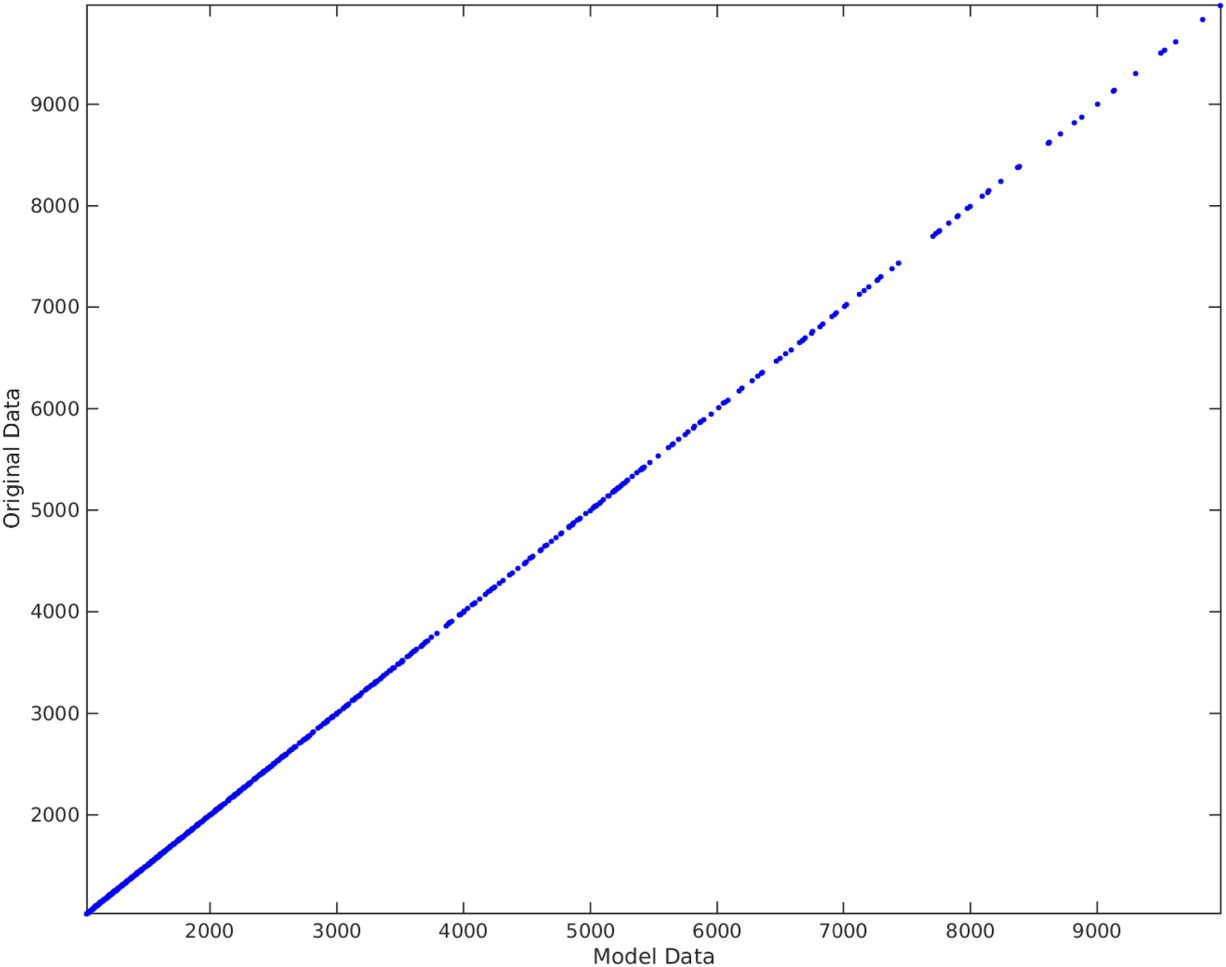
Algorithm 1 applied on $y=120+\frac{1000}{x_{2}}$. The horizontal axis shows the model found by our proposed method. The vertical axis shows the output *y*.

**Fig 8:**
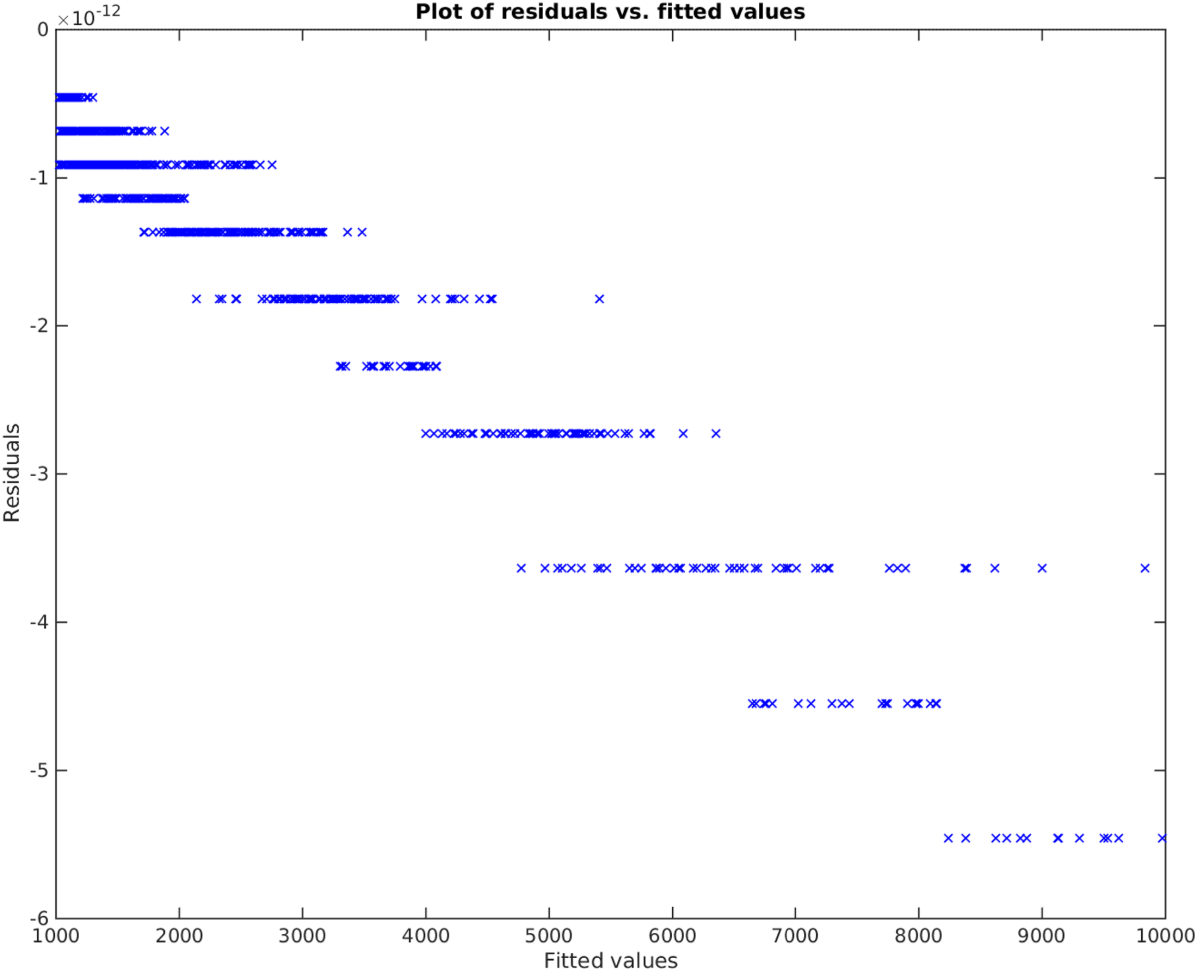
Residual plot of our proposed method applied on $y=120+\frac{1000}{x_{2}}$. Note that the vertical axis is of the order 10^−12^. The error perceived here is due to floating point and rounding error.

We sampled 1000 data points of *x*_1_, *x*_2_, and *x*_3_ and then created the non-linear functional $y=120+\frac{1000}{x_{2}}$. We take the original input matrix **X** to be composed of all *x*_1_, *x*_2_, and *x*_3_. Using the traditional best subset selection [7], accompanied with a control over *VIF* not to get above 10, we get the results shown in Fig 5. Again, from this figure, it is clear that the best subset selection model is not capable of capturing the correct non-linearity in the model. The heteroscedasticity of the residual plot can be seen in Fig 6. The found subset of parameters is {*x*_1_, *x*_2_}. On the other hand, if Algorithm 1 is used, the non-linearity is captured completely (See Figs 7 and 8). The subset of parameters found by our proposed method is the non-linear parameter $\left\{\frac{1}{x_{2}}\right\}$. Here, ς=0.8 and the default value of *δ* was used.

## Real data example

The synthetic examples in the previous section showed the capability of our method in capturing the true non-linearity of a dataset. In this section, we show a real data case study.

Cardiovascular Diseases (CVDs) are the major cause of deaths in the United States, killing more than 350,000 people every year [31]. One of the major contributors to CVDs is arterial stiffness [32, 33]. Arterial stiffness can be approximated by Carotid-femoral Pulse Wave Velocity (PWV) [34]. In fact, PWV is one of the most important quantitative index for arterial stiffness [33]. PWV measures the speed of the arterial pressure waves traveling along the blood vessels and higher PWV usually highlights stiffer arteries. Increased aortic stiffness is related to many clinically adverse cardiovascular outcomes [32]. PWV constitutes an independent and valuable marker for cardiovascular diseases (CVDs) and its use is crucial as a routine tool for clinical patient assessment.

In this section, our aim is not to present the most accurate PWV model. However, our goal is to show that if our technique of model construction is used (see Algorithm 1), we are able to find a more interpretable model.

The data we present is collected from 5444 Framingham Heart Study (FHS) participants [35]. Each participant had undergone an arterial tonometry data collection. The participants were part of FHS Cohorts Gen 3 Exam 1 [36], Offspring Exam 7 [37], and Original Exam 26 [38]. The California Institute of Technology and Boston University Medical Center Institutional Review Boards approved the protocol and all participants gave written informed consent. Here, we try to find models for PWV based on the following inputs: Age (*A*), Pulse Duration (*D*), Weight (*W*), Height (*H*), and Body Mass Index (*BMI*).

One model is based on the traditional best subset selection method monitored for *VIF* < 10, and the other based on the Parameter Selection Algorithm method (Algorithm 1). The participant characteristics are shown in Table 1.

**Table 1:** Participant characteristics.

	Range	Median
Duration	0.58 to 1.77	0.98
Age	19 to 99	46
Weight	83 to 339	165
Height	54.00 to 78.75	66.50
BMI	15.47 to 51.47	25.89
PWV	3.5 to 29.6	7.4

### Best subset selection model results

**Fig 9:**
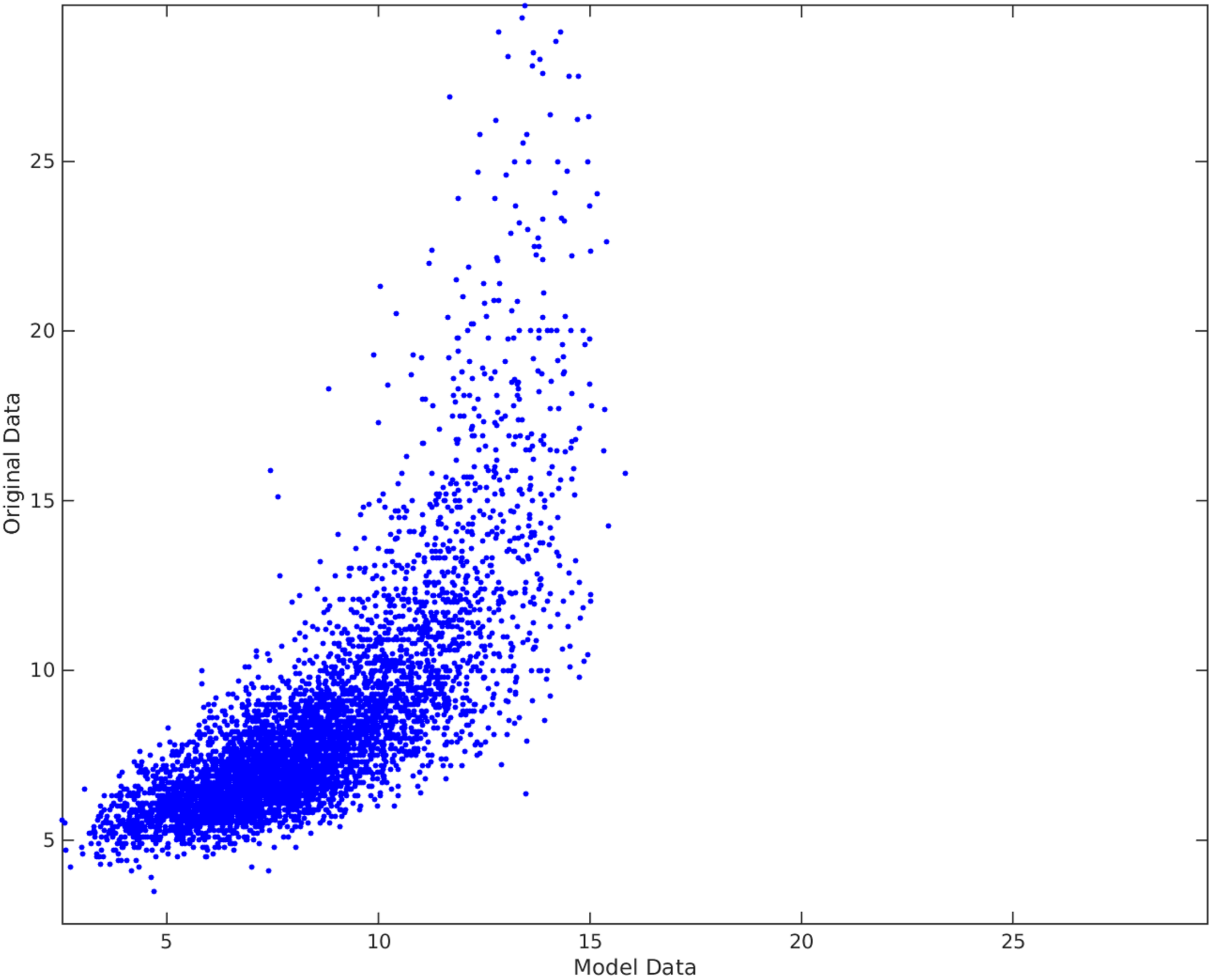
Traditional best subset selection method applied on PWV data. The horizontal axis shows the model found by the best subset selection method. The vertical axis shows the recorded PWV data. The $R_{adj}^{2}$ of the model is 0.56737.

**Fig 10:**
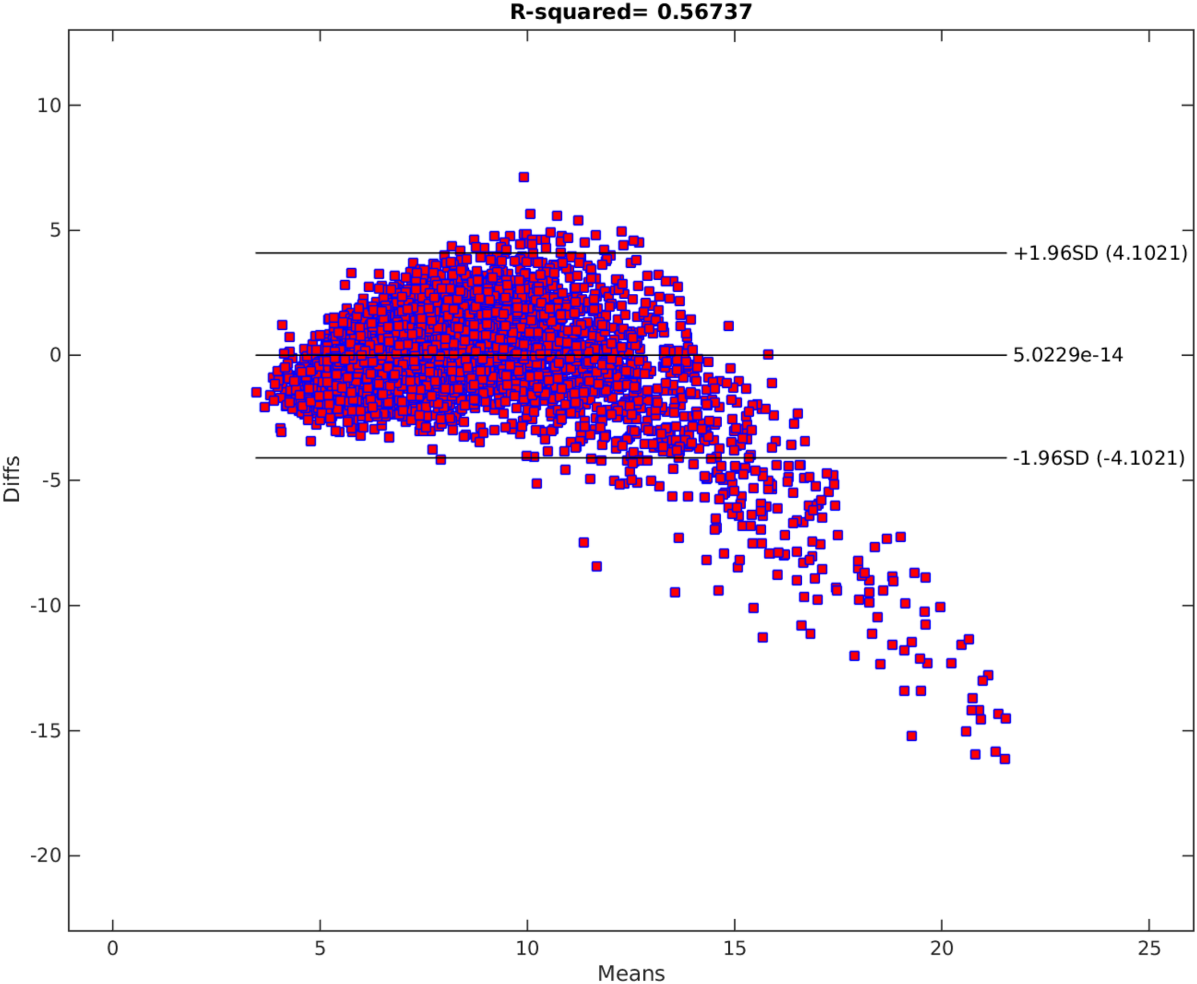
Bland-Altman of the traditional best subset selection model. The horizontal axis shows the means of the fitted and original PWV values. The Vertical axis shows the differences between the fitted and original PWV values.

**Fig 11:**
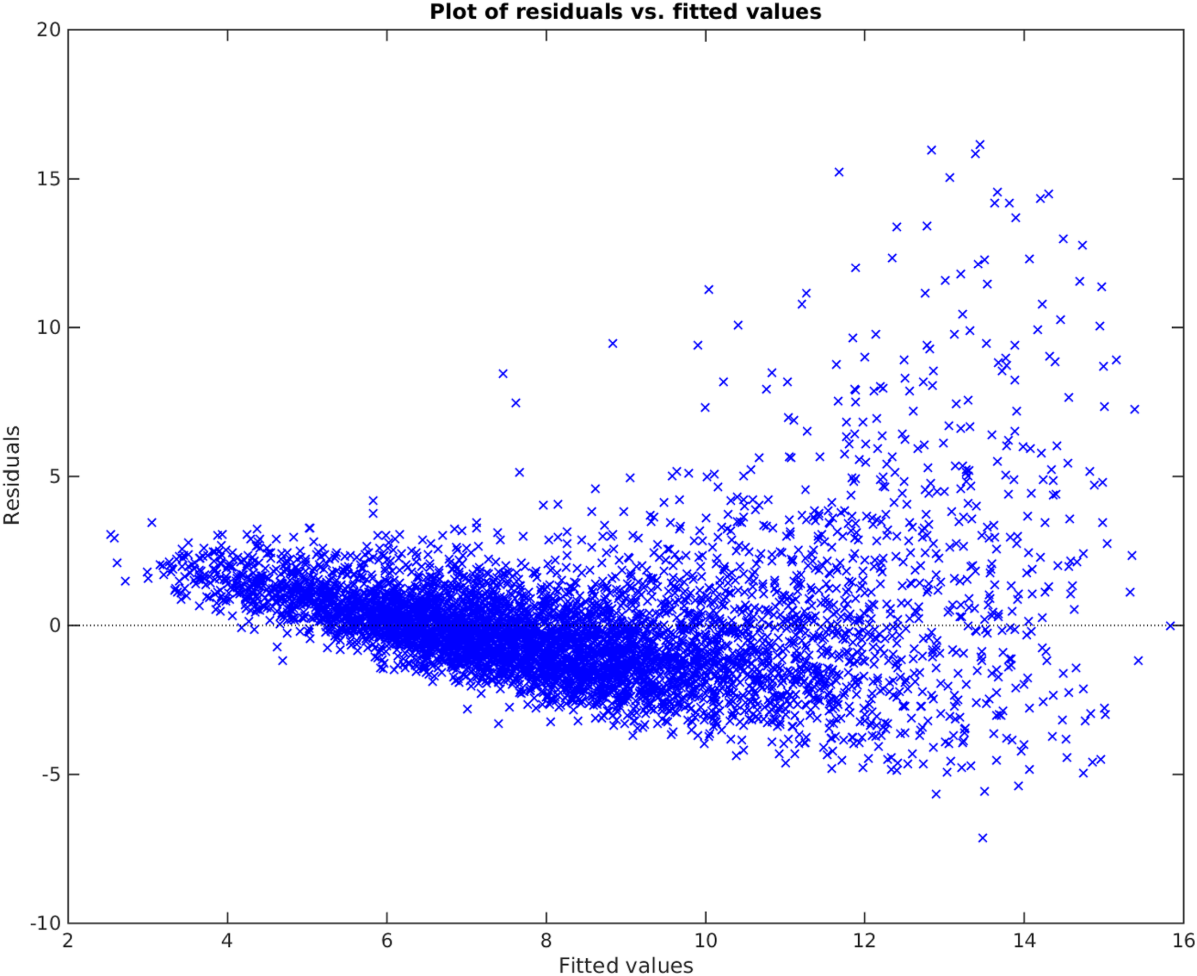
Residual plot of best subset selection method.

Fig 9 shows the traditional best subset selection method applied on PWV data. As seen in the plot, the best subset selection model cannot capture the non-linearity in the data set and completely misses the PWV values above 15. The heteroscedasticity of the residual can be seen from the Bland-Altman plot in Fig 10 and residual plot in Fig 11. The $R_{adj}^{2}$ of this model is 0.56737. The found subset of parameters is {*D*, *A*, *BMI*, *H*}. The p-value of these parameters are 3 × 10^−45^, 0, 2 × 10^−14^, and 1 × 10^−11^, receptively.

### Parameter selection algorithm results

**Fig 12:**
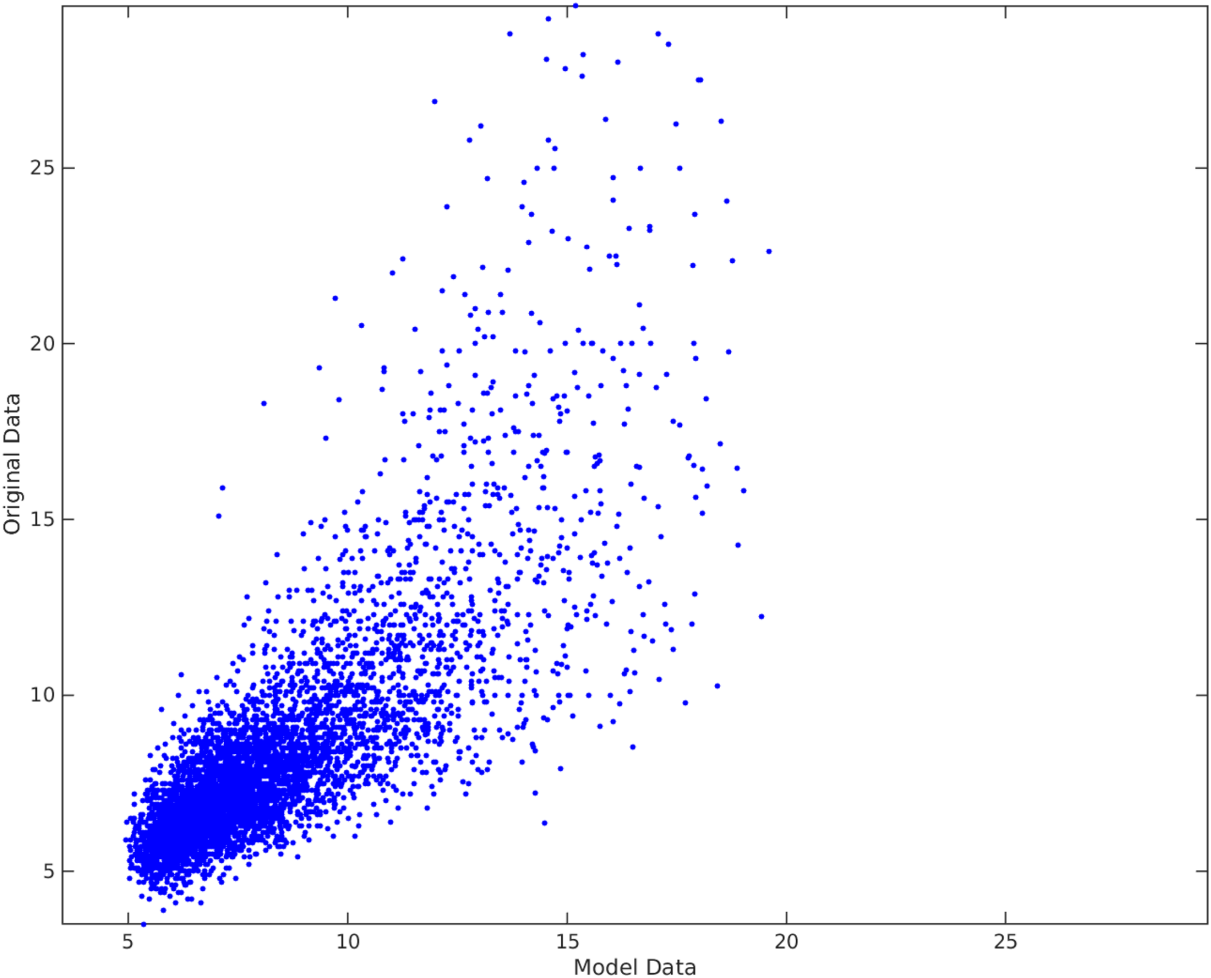
Parameter selection algorithm method applied on PWV data. The horizontal axis shows the model found by the best subset selection method. The vertical axis shows the recorded PWV data. The $R_{adj}^{2}$ of the model is 0.63052.

**Fig 13:**
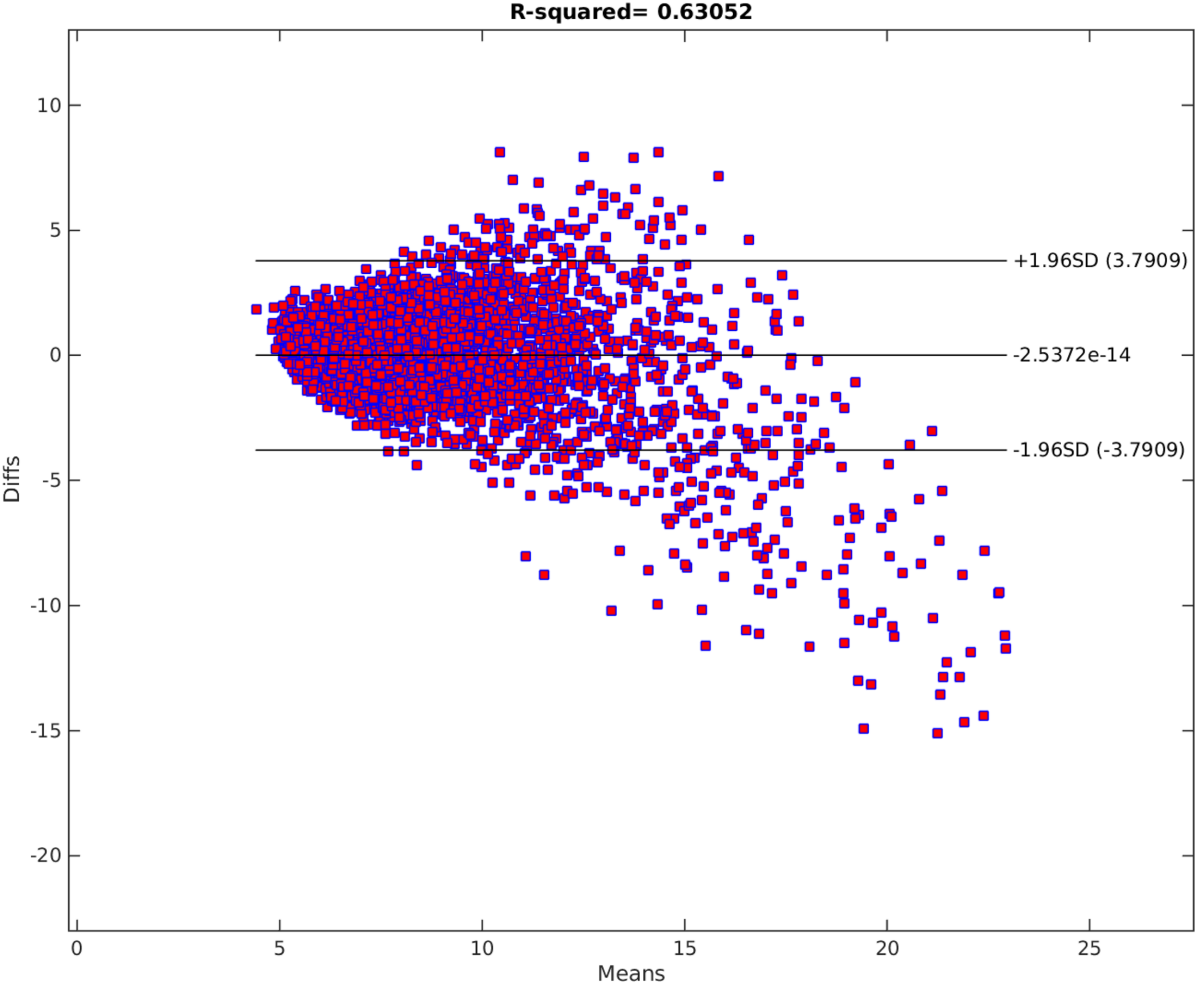
Bland-Altman of the parameter selection algorithm model. The horizontal axis shows the means of the fitted and original PWV values. The Vertical axis shows the differences between the fitted and original PWV values.

**Fig 14:**
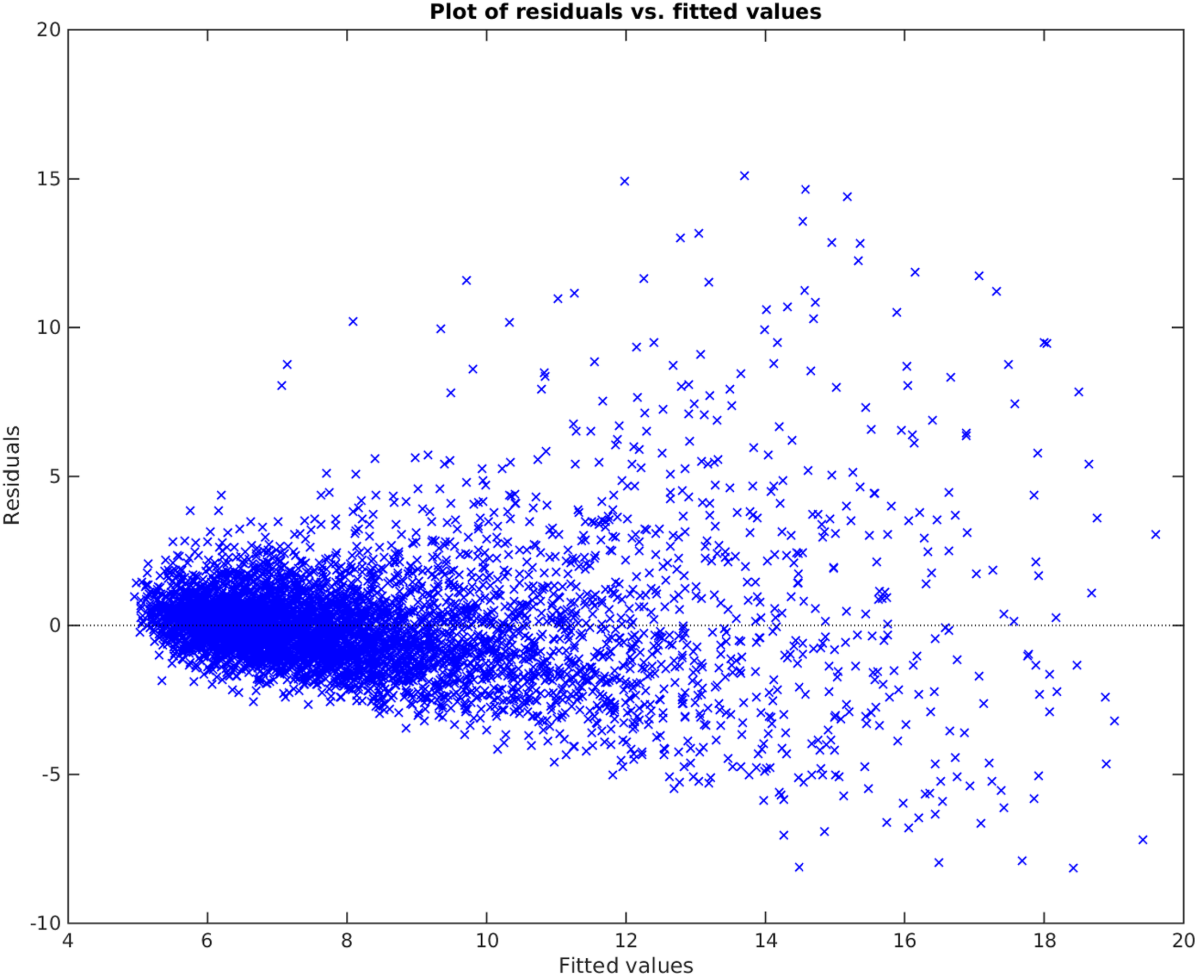
Residual plot of parameter selection algorithm.

Fig 12 shows the Parameter Selection Algorithm method applied on PWV data. Here, *δ* was the default value and ς=0.8. As seen on the plot, Parameter Selection Algorithm can fairly capture the non-linearity in the data set. The residuals can be seen in the Bland-Altman plot in Fig 13 and residual plot in Fig 14. The $R_{adj}^{2}$ of the model is 0.63052 (The correlation coefficient is 0.79). The found subset of parameters is

$$\begin{array}{c} \{DA,\frac{A^{2}}{\sqrt{D}},\frac{{\left(\log(A)\right)}^{2}}{{\left(\log(W)\right)}^{2}},\frac{{\left(\log(BMI)\right)}^{2}}{A}\}. \end{array}$$ (15)

The p-value of these parameters are 6 × 10^−4^, 0, 2 × 10^−21^, and 8 × 10^−46^, receptively.

From (15) one can interpret that Age (*A*) is a dominant factor in PWV. Furthermore, The Age adjustments with Heart Rate $HR=\frac{60}{D}$ is of great importance. The other interpretable factors are the adjusted values of slenderness (body mass index *BMI*, and the weight *W*) with respect to Age (*A*). Height (*H*) is not a factor of importance at all. As we can observe, the Parameter Selection Algorithm can provide an interpretable non-linear model of this critical physiological parameter.

### Comparison and results discussion

Comparing Figs 9 and 12, it is clear that the Parameter Selection Algorithm method is superior to the best subset selection method. The $R_{adj}^{2}$ of the Parameter Selection Algorithm model is almost %11 better than the best subset selection method. Both methods suffer in capturing all the variation and non-linearity in data (compare Fig 10 to Fig 13 and Fig 11 to Fig 14). However, Parameter Selection Algorithm is better in this respect. The heteroscedasticity of the best subset selection method is worse than that of the Parameter Selection Algorithm method (compare Fig 11 to Fig 14). The Bland-Altman limits of agreement of the Parameter Selection Algorithm method is also better than those of the best subset selection method (compare Fig 10 to Fig 13). The latter shows that the Parameter Selection Algorithm method is a more precise method than the best subset selection method.

### Comparison with neural networks

Although our purpose, in this article, is not to compete with state of the art statistical learning algorithms, we decided to compare our results with ANN. We provided the same input {*D*, *A*, *W*, *BMI*, *H*} to a neural network with five nodes. The output estimate of the neural network had a 0.81 correlation with the true values. Our method has a correlation coefficient of 0.79. Although the Parameter Selection Algorithm is designed mainly to act as a interpretable data mining method, it has a relatively acceptable accuracy. The 0.02 drop in correlation coefficient could possibly be neglected with the fact that, compared to neural networks, the non-linear output of the Parameter Selection Algorithm is interpretable and also behaves as a dimensionality reduction algorithm.

Finally, we again mention that our goal is not to show the best possible model for PWV, but rather to show the capabilities of our presented method.

### Other applications

The interpretability of Algorithm 1 would be an advantage in analyzing physiological data. In other words, complex biomedical and bioengineering databases could be appropriate fits for this method.

In previous section, we expressed the application of our algorithm to PWV data. This suggests that any continuous physiological variable can be treated the same way. For example, important biomedical continuous variables such as Cardiac Output (CO) [39], Ejection Fraction (EF) [40], Stroke Volume (SV) [39], Blood Pressure (BP), and Homeostatic Model Assessment (HOMA) [41] can all be estimated and interpreted using Algorithm 1. This list variables can be extended beyond the mentioned cases.

## Conclusion and future works

In this paper, we have introduced the Parameter Selection Algorithm (Algorithm 1) by which one can simultaneously capture some of the non-linearities of the data into the model, introduce automatic interpretable interaction and transformation among predictions, and also pick the best model. This approach minimizes the efforts done by an analyst and is virtually automatic. So far, up to the best of our knowledge, no other algorithm or method is able to perform these tasks at the same time automatically.

Here, our purpose has not been to introduce a competing statistical learning method, but to furnish a data mining tool. Despite this, we have shown that our model is almost as good as the state of the art statistical learning algorithms.

This data mining approach provides an interpretable dimensionality reduction model that faithfully models the data. We believe, the Parameter Selection Algorithm could have versatile applications in biostatistics as shown by one of the examples in this manuscript.

The hyper-parameters *ς* and *δ*, presented in this article, are analyzed and set heuristically. In a future work, we intend to perform a more detailed analysis to possibly quantify optimum values for them. Furthermore, instead of just solving step 5 in Algorithm 1, we could also add the constraint that parameters with high p-values, in a model, should be discarded to provide an even sparser result.

All in all, we see this article as a proof of concept which needs further investigation to analyze the involved hyper-parameters and also tweaks to its optimization core.

## Supporting information

S1 Dataset Example datasets. This file includes all synthetic data examples in this manuscript.
(ZIP)

## References

[1] Montgomery DC, Peck EA, Vining GG. Introduction to linear regression analysis. John Wiley & Sons; 2015.

[2] Friedman J, Hastie T, Tibshirani R. The elements of statistical learning. vol. 1. Springer series in statistics Springer, Berlin; 2001.

[3] Furnival GM. All possible regressions with less computation. Technometrics. 1971;13(2):403–408. doi: 10.1080/00401706.1971.10488794

[4] Garside M. The best sub-set in multiple regression analysis. Applied Statistics. 1965;p. 196–200.

[5] Morgan J, Tatar J. Calculation of the residual sum of squares for all possible regressions. Technometrics. 1972;14(2):317–325. doi: 10.1080/00401706.1972.10488918

[6] Schatzoff M, Tsao R, Fienberg S. Efficient calculation of all possible regressions. Technometrics. 1968;10(4):769–779. doi: 10.2307/1267458

[7] Furnival GM, Wilson RW. Regressions by leaps and bounds. Technometrics. 2000;42(1):69–79. doi: 10.1080/00401706.2000.10485982

[8] Efroymson M. Multiple regression analysis. Mathematical methods for digital computers. 1960;1:191–203.

[9] Mallows C. Choosing variables in a linear regression: A graphical aid. In: Central Regional Meeting of the Institute of Mathematical Statistics, Manhattan, Kansas. vol. 5; 1964.

[10] Mallows CL. More comments on Cp. Technometrics. 1995;37(4):362–372. doi: 10.2307/1269729

[11] Mallows CL. Some comments on Cp. Technometrics. 1973;15(4):661–675. doi: 10.1080/00401706.1973.10489103

[12] Mallows CL. Choosing a subset regression. In: TECHNOMETRICS. vol. 9. AMER STATISTICAL ASSOC 1429 DUKE ST, ALEXANDRIA, VA 22314; 1967. p. 190.

[13] Olusegun AM, Dikko HG, Gulumbe SU. Identifying the Limitation of Stepwise Selection for Variable Selection in Regression Analysis. American Journal of Theoretical and Applied Statistics. 2015;4(5):414–419. doi: 10.11648/j.ajtas.20150405.22

[14] Hoerl AE, Kennard RW. Ridge regression: Biased estimation for nonorthogonal problems. Technometrics. 1970;12(1):55–67. doi: 10.1080/00401706.1970.10488634

[15] Hoerl AE, Kennard RW. Ridge regression: applications to nonorthogonal problems. Technometrics. 1970;12(1):69–82. doi: 10.1080/00401706.1970.10488634

[16] García C, García J, López Martín M, Salmerón R. Collinearity: Revisiting the variance inflation factor in ridge regression. Journal of Applied Statistics. 2015;42(3):648–661. doi: 10.1080/02664763.2014.980789

[17] Chen SS, Donoho DL, Saunders MA. Atomic decomposition by basis pursuit. SIAM journal on scientific computing. 1998;20(1):33–61. doi: 10.1137/S1064827596304010

[18] Candes EJ, Tao T. Near-optimal signal recovery from random projections: Universal encoding strategies? Information Theory, IEEE Transactions on. 2006;52(12):5406–5425. doi: 10.1109/TIT.2006.885507

[19] Efron B, Hastie T, Johnstone I, Tibshirani R, et al Least angle regression. The Annals of statistics. 2004;32(2):407–499. doi: 10.1214/009053604000000067

[20] Candes E, Tao T. The Dantzig selector: Statistical estimation when p is much larger than n. The Annals of Statistics. 2007;p. 2313–2351.

[21] Barber RF, Candès EJ, et al Controlling the false discovery rate via knockoffs. The Annals of Statistics. 2015;43(5):2055–2085. doi: 10.1214/15-AOS1337

[22] Stone M, Brooks RJ. Continuum regression: cross-validated sequentially constructed prediction embracing ordinary least squares, partial least squares and principal components regression. Journal of the Royal Statistical Society Series B (Methodological). 1990;p. 237–269.

[23] Zou H, Hastie T, Tibshirani R. Sparse principal component analysis. Journal of computational and graphical statistics. 2006;15(2):265–286. doi: 10.1198/106186006X113430

[24] Trefethen LN, Bau III D. Numerical linear algebra. vol. 50. Siam; 1997.

[25] Box GE, Cox DR. An analysis of transformations. Journal of the Royal Statistical Society Series B (Methodological). 1964;p. 211–252.

[26] Box GE, Tidwell PW. Transformation of the independent variables. Technometrics. 1962;4(4):531–550. doi: 10.1080/00401706.1962.10490038

[27] MacKay DJ. Information theory, inference and learning algorithms. Cambridge university press; 2003.

[28] Hinton G, Deng L, Yu D, Dahl GE, Mohamed Ar, Jaitly N, et al Deep neural networks for acoustic modeling in speech recognition: The shared views of four research groups. IEEE Signal Processing Magazine. 2012;29(6):82–97. doi: 10.1109/MSP.2012.2205597

[29] Marquaridt DW. Generalized inverses, ridge regression, biased linear estimation, and nonlinear estimation. Technometrics. 1970;12(3):591–612. doi: 10.1080/00401706.1970.10488699

[30] Cohen J. A power primer. Psychological bulletin. 1992;112(1):155 doi: 10.1037/0033-2909.112.1.155 19565683 10.1037//0033-2909.112.1.155

[31] Association AH, et al. Heart Disease and Stroke Statistics–At-a-Glance; 2015.

[32] Mitchell GF, Hwang SJ, Vasan RS, Larson MG, Pencina MJ, Hamburg NM, et al Arterial stiffness and cardiovascular events the Framingham Heart Study. Circulation. 2010;121(4):505–511. doi: 10.1161/CIRCULATIONAHA.109.886655 20083680 10.1161/CIRCULATIONAHA.109.886655 PMC2836717

[33] Mitchell GF, Parise H, Benjamin EJ, Larson MG, Keyes MJ, Vita JA, et al Changes in arterial stiffness and wave reflection with advancing age in healthy men and women the Framingham Heart Study. Hypertension. 2004;43(6):1239–1245. doi: 10.1161/01.HYP.0000128420.01881.aa 15123572 10.1161/01.HYP.0000128420.01881.aa

[34] Safar ME, London GM, et al Therapeutic studies and arterial stiffness in hypertension: recommendations of the European Society of Hypertension. Journal of hypertension. 2000;18(11):1527–1535. doi: 10.1097/00004872-200018110-00001 11081763 10.1097/00004872-200018110-00001

[35] Framingham Heart Study;. Accessed: 2016-07-14. https://www.framinghamheartstudy.org/

[36] Splansky GL, Corey D, Yang Q, Atwood LD, Cupples LA, Benjamin EJ, et al The third generation cohort of the National Heart, Lung, and Blood Institute’s Framingham Heart Study: design, recruitment, and initial examination. American journal of epidemiology. 2007;165(11):1328–1335. doi: 10.1093/aje/kwm021 17372189 10.1093/aje/kwm021

[37] Kannel WB, Feinleib M, McNAMARA PM, Garrison RJ, Castelli WP. An investigation of coronary heart disease in families The Framingham offspring study. American journal of epidemiology. 1979;110(3):281–290. doi: 10.1093/oxfordjournals.aje.a112813 474565 10.1093/oxfordjournals.aje.a112813

[38] Dawber TR, Meadors GF, Moore FE Jr. Epidemiological Approaches to Heart Disease: The Framingham Study*. American Journal of Public Health and the Nations Health. 1951;41(3):279–286. doi: 10.2105/AJPH.41.3.279 10.2105/ajph.41.3.279 PMC1525365 14819398

[39] Geerts BF, Aarts LP, Jansen JR. Methods in pharmacology: measurement of cardiac output. British journal of clinical pharmacology. 2011;71(3):316–330. doi: 10.1111/j.1365-2125.2010.03798.x 21284692 10.1111/j.1365-2125.2010.03798.x PMC3045542

[40] Greupner J, Zimmermann E, Grohmann A, Dübel HP, Althoff T, Borges AC, et al Head-to-head comparison of left ventricular function assessment with 64-row computed tomography, biplane left cineventriculography, and both 2-and 3-dimensional transthoracic echocardiography: comparison with magnetic resonance imaging as the reference standard. Journal of the American College of Cardiology. 2012;59(21):1897–1907. 22595410 10.1016/j.jacc.2012.01.046

[41] Matthews D, Hosker J, Rudenski A, Naylor B, Treacher D, Turner R. Homeostasis model assessment: insulin resistance and *β*-cell function from fasting plasma glucose and insulin concentrations in man. Diabetologia. 1985;28(7):412–419. doi: 10.1007/BF00280883 3899825 10.1007/BF00280883

